# LC-MS/MS Method for Rapid Quantification of Progesterone in Rabbit Plasma and Its Application in a Pharmacokinetic Study of the Transdermal Formulation

**DOI:** 10.1155/2020/8889375

**Published:** 2020-10-30

**Authors:** Cao-Son Tran, Quang-Dong Bui, Ngoc-Tho Thi Nguyen, Minh-Hanh Dao, Thach-Tung Nguyen

**Affiliations:** ^1^Laboratory of Food Toxicology and Allergens Testing, National Institute for Food Control, Hanoi 10000, Vietnam; ^2^Department of Pharmaceutics, Hanoi University of Pharmacy, Hanoi 10000, Vietnam

## Abstract

A rapid and effective method using QuEChERS-based sample preparation procedure and liquid chromatography-tandem mass spectrometry (LC-MS/MS) analysis has been developed and validated to determine progesterone in rabbit plasma. The analyte was extracted from plasma by acetonitrile with phase partitioning by a mixture of magnesium sulfate and sodium chloride. The supernatant was then directly injected into LC-MS/MS in a positive electrospray ionization mode and quantified using progesterone-d9 as the internal standard. The method linearity was in the range from 1 ng/mL (LOQ) to 200 ng/mL. Method recovery was from 86.0% to 103%, and repeatability was lower than 5.5%. The plasma sample was stable for 12 weeks stored at 18 ± 2°C. This method was applied to quantify progesterone in rabbit plasma in a pharmacokinetic study of two transdermal formulations: a reference drug and a eutectic-hydrogel system. The data indicate that the eutectic-hydrogel system's bioavailability was 1.5 times better than that of the reference drug, and the transdermal system is a potential drug delivery system for progesterone.

## 1. Introduction

**Figure 1:**
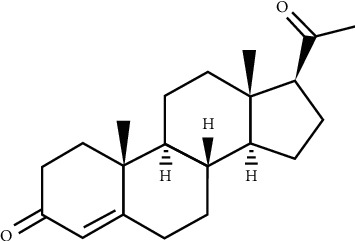
Chemical structure of progesterone.

Progesterone (Figure 1) is an endogenous steroid hormone secreted from the ovaries, testes, adrenal cortex, and placenta. It is the most critical hormone of progestins since this chemical has a crucial impact on the development of the uterus, fallopian tube, and breast [1]. Progesterone promotes cells to proliferate, enlarge, and become a secretary in nature [2]. The average serum progesterone level in adult women ranges from 0.15 to 25 ng/mL, but it can reach 150 ng/mL during pregnancy [3, 4]. This level in men and postmenopausal women was 0.38 ± 0.13 ng/ml and 0.38 ± 0.37 ng/mL, derived from a study of Winkelmann et al. [5]. Progesterone has been indicated to contraception, implantation, breast cancer, autoimmune, or progesterone deficiency diseases [6–9]. The transdermal formulations of progesterone have been developed for contraception, breast cancer, and artificial insemination support [7, 10, 11].

Some pharmacokinetic studies have evaluated the effectiveness of various formulations of progesterone [12–14], including the transdermal application [11]. Fraser et al., in an initial pharmacokinetic trial, evaluated the progesterone level in volunteers' blood after applying Nestorone®, a spray formulation of progesterone. The study indicated that progesterone's serum level to block ovulation was achievable, and it can provide effective contraception [11]. However, the evidence which proves the transdermal application of progesterone is inadequate [10]. There is a need to study the progesterone's pharmacokinetics in the transdermal gel to evaluate its efficacy and safety.

Many methods have been used for analyzing progesterone in plasma. A conventional method for progesterone analysis is radioimmunoassay (RIA). Abraham et al. developed an RIA method to determine progesterone in plasma: the sensitivity varied from 10 to 25 pg, and the recovery was 84.2 ± 4.8% [15]. Two enzyme immunoassay (EIA) methods were developed using two different enzymes, horseradish peroxidase (HP) and alkaline phosphatase (AP), and were compared with RIA. The EIA-HP method's precision was comparable with the RIA method, and the detection limit was 10 times better than RIA. The EIA-AP method was not suitable to determine progesterone in the plasma because the value of this assay was three times higher than those measured by the other methods. Although RIA and EIA methods have their pros for high specificity and sensitivity, its cons are present in complexity in its cost and instruments [16].

Liquid chromatography-tandem mass spectrometry (LC-MS/MS) has recently become a widely used method to determine progesterone in biological matrices. Tai et al. developed a liquid-liquid extraction followed by LC-MS/MS analysis to quantify progesterone in human serum. Progesterone and the internal standard (progesterone-^13^C2) were duplicate extracted into *n*-hexane. The extract was then dried under nitrogen and reconstituted in methanol containing acetic acid before directly injected into LC-MS/MS. The method was successfully validated in the range from 0.151 to 24.42 ng/g [17]. Fernandes et al. applied a similar procedure in cattle plasma using medroxyprogesterone acetate as the internal standard and compared it to the RIA method. The LC-MS/MS method gave a higher progesterone concentration than the RIA method, explaining that the RIA method is affected by interferences in the matrix [18]. Zhang et al. used solid-phase extraction with Oasis HLB cartridge prior to LC-MS/MS analysis to determine 17*α*-hydroxyprogesterone caproate, 17*α*-hydroxyprogesterone, and progesterone in human plasma with medroxyprogesterone acetate as the internal standard. The linearity of progesterone was from 1 to 200 ng/mL [14]. Sasaki et al. employed salting-out assisted liquid-liquid extraction with LC-MS/MS for progesterone analysis to obtain easier and quicker sample preparation steps. With ammonium acetate as the salting-out agent, this method was applied to analyze progesterone in rat plasma from 0.05 to 20 ng/mL. The heavy matrix effect was controlled using the calibration curve on the surrogate matrix (water) [13].

Plasma analysis requires a method that is high throughput and appropriate for a limited amount of plasma. QuEChERS methodology, invented by Anastassiades and Lehotay for multiresidue analysis of pesticides [19], has been applied in extracting various pharmaceutical compounds in plasma matrices [20–23]. Intending to develop a method that is quick and uses less solvent as well as to evaluate the pharmacokinetic study of progesterone from the transdermal route, this paper presents a rapid QuEChERS-based method coupled with LC-MS/MS for the quantification of progesterone in rabbit plasma.

## 2. Materials and Methods

### 2.1. Standards, Reagents, and Materials

Progesterone analytical standard and isotope internal standard (progesterone-d9) were purchased from Sigma-Aldrich (St. Louis, MO, USA). Acetonitrile, ethanol, formic acid, ammonium chloride, anhydrous magnesium sulfate, and sodium chloride were obtained from Merck Vietnam (Hanoi, Vietnam). Ultrapure water was produced by a water filtration system (Milli-Q® Integral, Merck, Germany).

The progesterone and progesterone-d9 stock solutions, both of 100 *µ*g/mL, were separately prepared in ethanol. The stock solutions were then diluted with acetonitrile to the concentrations of 1 *µ*g/mL. The matrix-matched calibration curve was prepared in the blank extract with the progesterone concentration in the range of 1 to 200 ng/mL and progesterone-d9 concentration of 50 ng/mL.

The eutectic hydrogel of progesterone (EHP) was prepared in the Department of Pharmaceutics, Hanoi University of Pharmacy (obtained from another study). The reference drug formulation (RDF) was 1% progesterone gel (Besins Manufacturing, Belgium), which was purchased in the market.

### 2.2. Plasma Preparation

Rabbit plasma was brought to room temperature, and 500 *µ*L was pipetted into 2 mL centrifuged tube followed by the addition of 25 *µ*L internal standard solution of 1 *µ*g/mL and 475 *µ*L of acetonitrile. After being vigorously shaken by a vortex mixer, the tube received a mixture of salts and sorbents (described below), and it was mixed thoroughly for 1 min. The tube was then centrifuged at 13,000 rpm in 5 mins, and the supernatant was passed through a 0.2 *µ*m PTFE filter and analyzed by LC-MS/MS.

The composition of salting-out and cleaning agents will be accessed by comparing the efficiency of different mixtures of salts and sorbents: (1) 200 mg of MgSO_4_, (2) 150 mg of MgSO_4_ and 50 mg of NaCl, (3) 150 mg of MgSO_4_ and 50 mg of PSA, (4) 150 mg of MgSO_4_ and 50 mg of C18, (5) 200 mg of CH_3_COONH_4_, and (6) 200 mg of NH_4_Cl.

The blank sample was extracted via the abovementioned procedure without adding IS in the first step. The final extract was used to prepare working standard solutions at the concentration from 1 to 200 *µ*g/mL (for progesterone) and 50 *µ*g/mL (for progesterone-d9).

### 2.3. Instrumentation

An LC 1290 coupled with a 6460 QQQ mass spectrometer (Agilent, USA) was used to determine progesterone and progesterone-d9. The analytes were separated in Eclipse plus Agilent XD8 C18 column (150 × 2.1 mm, 3.5 *µ*m particle size) with the mobile phase of deionized water (A) and acetonitrile (B); both contained 0.1% of formic acid, at the flow rate of 0.5 mL/minute. The gradient program was initially set at 10% B in 2 minutes. After that, the eluent composition gradually increases to 90% B in 2.5 minutes and maintains 4 minutes before returning to 10% in 1 minute. The system was finally reequilibrated in 2 minutes before the next injection.

The electrospray ionization source was operated in the positive mode to select the precursor ion. The collision energies were optimized to obtain the most abundant product ions. Two transitions of each compound were observed, of which the product mass with a higher intensity was chosen for the quantitative purpose, and the other mass was used for the confirmation purpose.

### 2.4. Method Validation

The method was validated according to the guideline for the bioanalytical method validation of the US FDA [24]. The specificity was assessed by comparing the chromatograms of the blank sample, the standard solution of 50 *µ*g/mL, and the blank sample spiked with progesterone and progesterone-d9 at the concentration of 50 *µ*g/mL. The noise ratio (S/N) methodology was applied to estimate the limit of detection (LOD) and limit of quantification (LOQ). The progesterone levels in blank spiked samples having S/N ratios of 3 and 10 were LOD and LOQ, respectively. Repeatability and recovery were evaluated by analyzing 6 replicates of spiked samples of three concentration levels: low-quality control (LQC) at 1 *µ*g/mL, medium-quality control (MQC) at 50 *µ*g/mL, and high-quality control at 200 *µ*g/mL.

Because of the low photochemical stability of progesterone, the long-term stability of the plasma sample was evaluated following the recommendation of the US FDA [24]. The blank rabbit plasma samples were spiked with progesterone at 2 concentrations of LQC and HQC. The first lot was analyzed in 6 replicates to determine the initial concentration. The remaining lots were stored at −18°C ± 2°C and were tested after 1 week, 2 weeks, 4 weeks, 8 weeks, and 12 weeks. The bias of the average concentration of each lot to the initial concentration should be within 15%.

### 2.5. Application in Pharmacokinetic Study

The animal study was approved by the Scientific and Ethics Committee, Hanoi University of Pharmacy. Male rabbits of about 2 kg each, purchased from the Centre of Experimental Animals, National Institute of Hygiene and Epidemiology (Hanoi, Vietnam), were selected for pharmacokinetic study. Male rabbits were chosen for pharmacokinetic experiments to avoid high fluctuating progesterone levels in female individuals. They were divided into two groups of three: one group for the reference drug and the other for the eutectic hydrogel system containing progesterone. Each gel (2.5 g) was applied to skin-free fur (5 cm × 10 cm) on the back of the rabbits_._ The blood samples (2 mL) were collected before applying the drug and at 4 hours, 6 hours, 7 hours, 9 hours, and 10 hours after the administration into an EDTA-coated tube. The tubes were centrifuged at 6000 rpm in 10 minutes, and the plasmas were collected and stored in 2 mL tubes at −10C before being injected into LC-MS/MS.

The Phoenix 8 software was used to calculate the pharmacokinetic parameters, including the maximum plasma concentration (*C*_max_), the time until *C*_max_ is reached (*T*_max_), and the area under the curve from time zero to ten hours (AUC0-10 h).

## 3. Results and Discussion

### 3.1. Optimization of Mass Spectrometry Condition

The ion transitions of progesterone and progesterone-d9 were obtained by directly infusing the standard solution of 1 *µ*g/mL into the mass spectrometer. The precursor ions were obtained when the molecular ion combined with a proton. The optimal collision energies were selected for two product ions (Table 1). Other mass spectrometer parameters were selected to gain the highest intensities of the analytes. Under these conditions, progesterone and progesterone-d9 peaks, both with a retention time of around 4.9 min, were symmetric and sufficient to the analysis (Supplement Figure S1).

### 3.2. Selection of Salting-Out Agents

**Figure 2:**
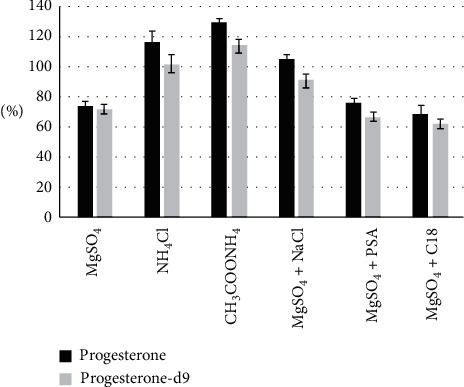
Progesterone and progesterone-d9 extraction recovery of different salting-out agents.

The average intensities of peak area in spiked samples (*n* = 2) and working standard solutions of the same concentration (50 *µ*g/mL) were compared to select the most effective partitioning and cleaning mixtures. The results are introduced in Figure 2.

The use of CH_3_COONH_4_ and NH_4_Cl gave the highest recovery of progesterone and progesterone-d9. However, the values of higher than 100% recovery indicate that the two layers are not completely separated: the amount of acetonitrile layer was less than the aqueous layer. The recovery when using MgSO_4_ was low because some water might still be in the acetonitrile layer. Although the salting-out assisted liquid/liquid extraction (SALLE), described by Sasaki et al. [13], used CH_3_COONH_4_ as a salting-out agent, this study showed that using CH_3_COONH_4_ will result in incomplete separation between aqueous and organic phase.

The QuEChERS extraction consists of two steps: first, the compound is usually extracted into acetonitrile from the water phase with the help of salting-out agents, and second, the extract is cleaned up by dispersive solid-phase extraction. Since the amount of plasma is limited, the second step was omitted, and the sorbents were added into the first step to investigate the cleaning efficiency. However, neither PSA nor C18 sorbent helped increase the recovery of progesterone. The combination of MgSO_4_ and NaCl gave the best extraction recovery of 105% and 91% for progesterone and progesterone-d9, respectively, and was chosen to be the salting-out agents in this extraction procedure.

### 3.3. Method Validation

The method specificity was accessed by comparing chromatograms of the blank sample, spiked sample at 50 ng/mL, and standard solution of 50 ng/mL (data shown in Supplements). There is no interference in the blank sample compared to the progesterone peak in the spiked sample and standard solution. Furthermore, the specificity was also supported by accessing the ion ratios of progesterone and progesterone-d9 (both about 100%) of the samples to those of the standards (Supplement Figure S1).

**Figure 3:**
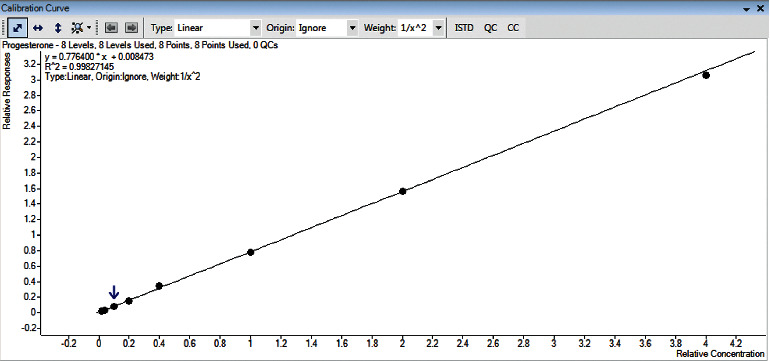
The calibration curve of progesterone on blank matrix.

LOD and LOQ were determined by calculating the S/N ratio in low-concentration spiked sample analysis (Supplement Figure S2). The LOD and LOQ were at 0.3 and 1 ng/mL, respectively, which were low enough for determining progesterone concentration in rabbit plasma. The LOD of the method was not as low as that of Sasaki's study [13], but it is fit for the purpose of analyzing the level of progesterone in plasma, which is usually higher than 1 ng/mL. The method was linear from 1 to 200 ng/mL with the coefficient of determination (*R*^2^) being higher than 0.99 (Figure 3).

The repeatability and recovery of progesterone at three concentrations (*n* = 6) are presented in Table 2. The method is precise and accurate, with the relative standard deviation lower than 5.5% and the recovery from 86.0% to 103%. This method was proven to meet the US FDA's requirements, had very high throughput (10 minutes to complete a set of 6 samples), and was environmental friendly (less than 0.5 mL of solvent for one extraction). These results indicate that the method can be a useful tool for pharmacokinetics studies of progesterone.

**Figure 4:**
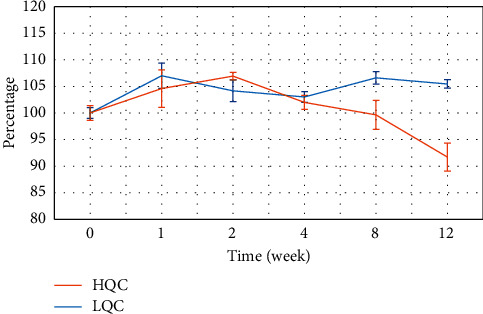
The stability of plasma sample spiked at two levels (LQC and HQC) within 12 weeks stored at −18°C ± 2°C.

The stability of the plasma sample stored at 18°C ± 2°C within 12 weeks is introduced in Figure 4. Through 12

weeks, the difference in concentration of the analyte in the plasma and the original concentration sample had not exceeded 15% for both HQC and LQC levels. The RSD value between the quantitative concentrations of each QC batch at the time of analysis was less than 15%. These results demonstrate that the plasma sample is stable for at least 12 weeks with the proper storage condition.

### 3.4. Pharmacokinetic Study

**Figure 5:**
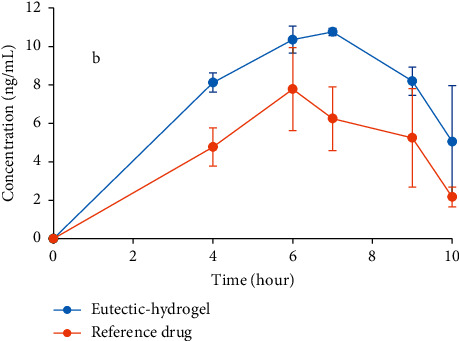
Progesterone content in rabbit plasma after applying the transdermal gels.

The *T*_max_ of the reference drug and the eutectic-hydrogel system (Table 3) was similar (6.33 hours and 6.67 hours) following the drug's pharmacokinetics through the skin: it takes several hours to reach the peak in plasma. This is because the drug has to undergo dissolution and absorption through the skin followed by the distribution, metabolism, and elimination process. The *C*_max_ of the eutectic-hydrogel system was higher than that of the reference drug, but it was not statistically different (*p*=0.13). Relative bioavailability data showed that the area under the curve from time zero to ten hours (AUC_0−10 h_) of the eutectic-hydrogel system was about 1.5 times higher than that of the reference drug (Table 3 and Figure 5).

The pharmacokinetics of progesterone on the rabbit has not yet been reported before. Compared with the study on volunteers of Fraser [11], the T_max_ of this study (6.33 to 6.67 hours) was lower than that of the single-dose application (20 hours) but higher than that of the multiple-dose treatment (4 hours) of a spray formulation. Because of the pharmacokinetic differences between species, the *C*_max_ value was incomparable.

This pharmacokinetic study may be affected by the limited number of rabbits used in this study. The standard deviations of some points in the pharmacokinetic curve of three cases were high (Figure 5), and it may change the actual values of pharmacokinetic parameters. Future studies may be needed with a larger number of objects and a longer time of sample collection. However, the trend of the curves is unaffectable, these results substantiate the transdermal gel of progesterone, and this formulation can be a potential route for future progesterone application.

## 4. Conclusions

We have validated a rapid and effective QuEChERS-based method to determine progesterone in rabbit plasma using liquid chromatography-tandem mass spectrometry. The method uses less organic solvent than conventional liquid-liquid extraction or solid-phase extraction methods and has suitable sensitivity and accuracy to quantify the progesterone concentration in plasma. The progesterone level in plasma was stable within 12 weeks of evaluation. The pharmacokinetics study showed a similar pattern of the pharmacokinetics of two transdermal formulations, and the eutectic-hydrogel system is proven to be a potential application of progesterone.

## Figures and Tables

**Table 1:** Multiple reaction monitoring conditions of progesterone and progesterone-d9.

Analytes	Precursor ion (m/*z*)	Product ion (m/*z*)	Collision energy, eV
Progesterone	315.2	109^a^	30
		97	26

Progesterone-d9	324.3	100^a^	30
		113	22

^a^Quantitative ion.

**Table 2:** Repeatability and recovery of progesterone at different levels.

Spiked level (ng/mL)	RSD (%)	R (%)
1	5.5	86.0–100
50	1.7	98.6–103
200	2.8	97.3–102

**Table 3:** Pharmacokinetic parameters of progesterone on rabbit models.

Parameter	Eutectic-hydrogel system (±SD)	Reference drug formulation (±SD)
*C*_max_ (ng/mL)	11.1 ± 0.66	8.49 ± 3.32
*T*_max_ (h)	6.67 ± 0.58	6.33 ± 0.58
AUC_0−10 h_ (ng.h/mL)	69.0 ± 6.49	46.5 ± 21.9

## Data Availability

The main part of the research data is included in the article. The chromatograms are included in the supplement file. Other data can be made available from the corresponding author upon request.

## Supplementary Materials

Supplementary Materials Figure S1: the chromatograms of standard solution of progesterone and progesterone-d9 (both of 50 ng/mL) with two ion transitions for each compound. Figure S2: chromatograms of progesterone in (a) working standard solution of 50 ng/mL, (b) blank sample spiked at 50 ng/mL, (c) blank sample, and (d) blank sample spiked at 1 ng/mL (LOQ).
