# Supplementary material for: LC-MS/MS Method for Rapid Quantification of Progesterone in Rabbit Plasma and Its Application in a Pharmacokinetic Study of the Transdermal Formulation
Source: J Anal Methods Chem. 2020 Oct 30;2020:8889375. doi: 10.1155/2020/8889375 (PMC7647766; doi:10.1155/2020/8889375)
Supplement: Supplementary Materials — Figure S1: the chromatograms of standard solution of progesterone and progesterone-d9 (both of 50 ng/mL) with two ion transitions for each compound. Figure S2: chromatograms of progesterone in (a) working standard solution of 50 ng/mL, (b) blank sample spiked at 50 ng/mL, (c) blank sample, and (d) blank sample spiked at 1 ng/mL (LOQ). [file 8889375.f1.docx]

**Supplement figures**


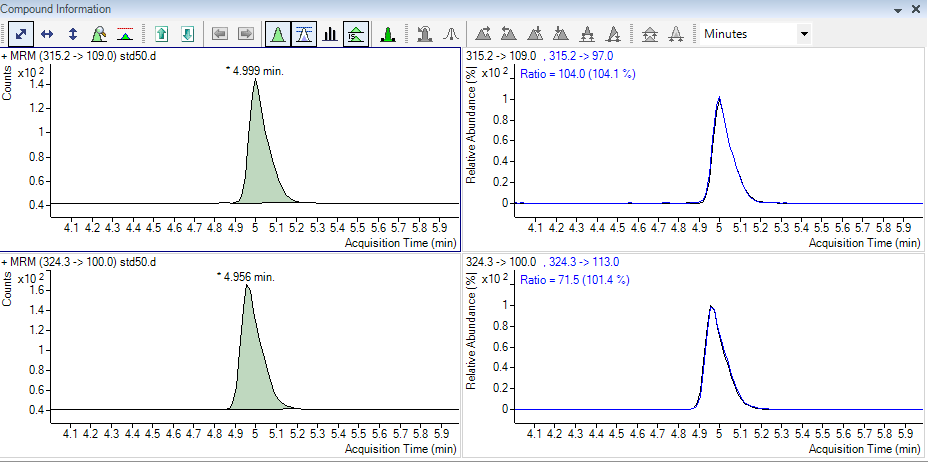


Figure S1: The chromatograms of standard solution of progesterone and progesterone-d9 (both of 50 ng/mL) with two ion transitions for each compound


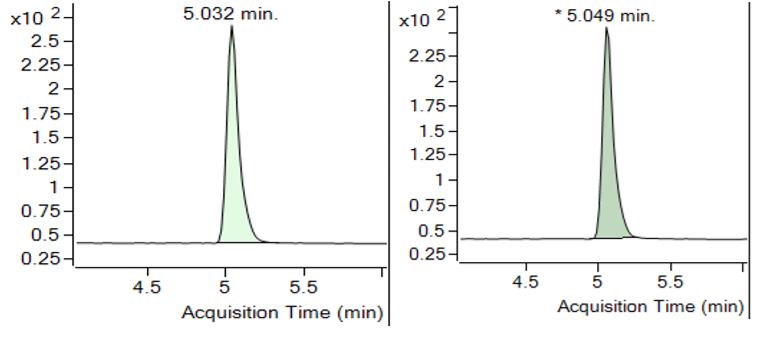


**a**

**b**


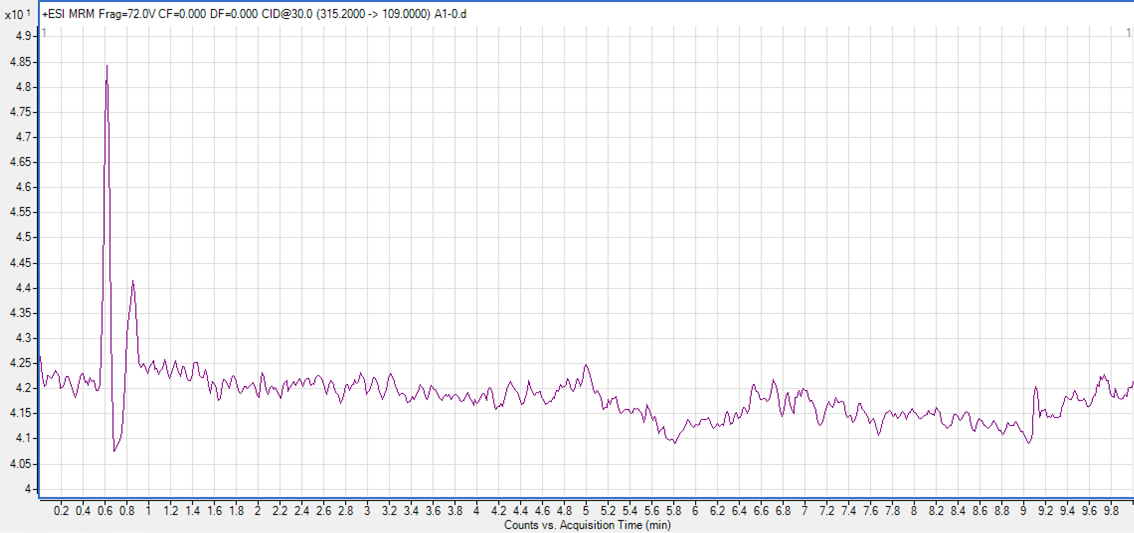

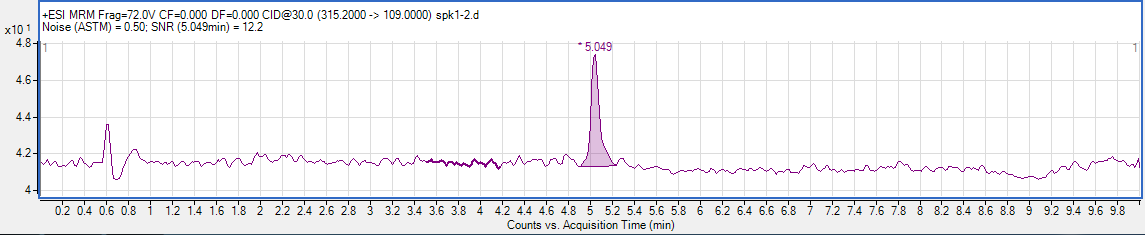


**d**

**c**

Figure S2: Chromatograms of progesterone in (a) working standard solution of 50 ng/mL and (b) blank sample spiked at 50 ng/mL, (c) blank sample and (d) blank sample spiked at 1 ng/mL (LOQ)
